# Giant Plexiform Schwannoma on the Medial Aspect of the Left Thigh

**DOI:** 10.7759/cureus.50136

**Published:** 2023-12-07

**Authors:** Amit Kumar, Sourabh Singh, Raghvendra P Singh, Ajeet P Maurya, Sramana Mukhopadhyay

**Affiliations:** 1 Medicine, All India Institute of Medical Sciences, Bhopal, IND; 2 General Surgery, All India Institute of Medical Sciences, Bhopal, IND; 3 Pathology and Laboratory Medicine, All India Institute of Medical Sciences, Bhopal, IND

**Keywords:** swelling of the thigh, antoni a and antoni b areas, giant, schwann cells, plexiform schwannoma

## Abstract

Plexiform schwannoma (PS), or neurilemmoma, is an uncommon benign tumor originating from a peripheral nerve sheath. It consists of Schwann cells organized in an intricate, web-like pattern. A male farmer in his 50s from rural India sought medical attention for a painless mass on his left thigh, present for 30 years. Physical examination revealed a firm, non-tender mass with restricted mobility. Imaging, including X-ray and ultrasound, indicated a neoplastic lesion. Fine needle aspiration (FNA) cytology revealed spindle-shaped cells, prompting a provisional diagnosis of a spindle cell lesion. Surgical excision was performed successfully, with histopathological examination confirming PS. He experienced no postoperative complications, and at the one-year follow-up, the complete resolution of symptoms and normal daily activities were observed.

## Introduction

Plexiform schwannoma (PS), or neurilemmoma, is an uncommon benign tumor originating from a peripheral nerve sheath. It consists of Schwann cells organized in an intricate, web-like pattern [1]. The tumor primarily arises from Schwann cells' slow and progressive multiplication [2]. Schwannomas constitute 5% of all soft-tissue tumors, with over half predominantly found in the head and spine [3]. Their occurrence in the extremities, particularly the thighs, is infrequent. It primarily affects individuals aged 30-40 years and typically appears as a solitary, slow-growing, painless nodule in the skin or subcutaneous tissue [4].

This case report presents the clinical details of a solitary PS diagnosed in an adult patient.

## Case presentation

We present a case involving a male farmer in his 50s from rural India who sought medical attention at our outpatient department (OPD). He reported a painless, progressively enlarging mass on the medial side of his left thigh over the past 30 years. The patient described the mass as non-tender, with no history of discharge or bleeding since its onset. Notably, there were no skin lesions, eye lesions (cataracts or nodules), or similar swellings elsewhere in the body. The patient had no significant medical history or prior surgeries but had been a smoker for the past 26 years. There was no family history of inherited disorders, swellings, or skin lesions. His biochemical and hematological parameters fell within the normal range.

**Figure 1:**
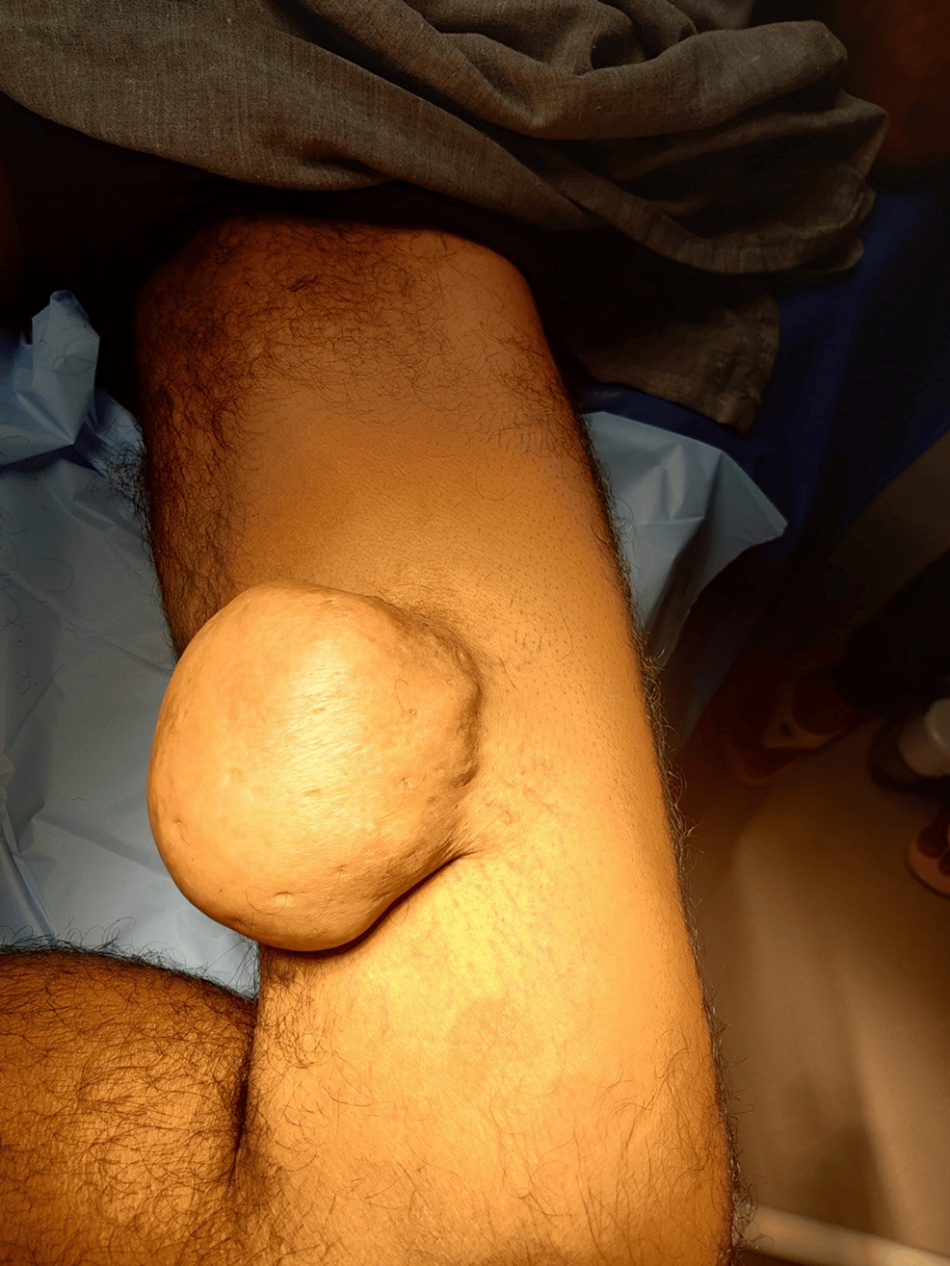
Swelling of the thigh measuring approximately 10 × 10 cm

Upon physical examination, a sizable, firm, pedunculated, and non-tender mass was palpable in the medial aspect of the left thigh, measuring approximately 10 × 10 cm (Figure 1).

The mass exhibited fixation to the underlying structures, with restricted mobility. The overlying skin appeared intact, showing no signs of discoloration or thickening. No muscle weakness was observed, and no sensation loss was noted in the affected area.

The X-ray of the left thigh indicated the absence of bony involvement, suggesting that the swelling originated from the soft tissues. Subsequent ultrasonography revealed a substantial lobulated, heterogeneous, solid cystic lesion measuring 10 × 9.2 × 6 cm. The lesion exhibited significant internal vascularity and was located in the medial aspect of the left thigh. The pedunculated lesion involved the skin and subcutaneous plane, with no observed deeper extension into the underlying muscular plane. These features raised the suspicion of a neoplastic lesion.

Following the ultrasound findings, fine needle aspiration (FNA) cytology was performed. Microscopic examination revealed clusters of spindle-shaped cells characterized by oval to elongated nuclei, unremarkable chromatin, moderate cytoplasm, and indistinct cell borders. Some groups exhibited mild to moderate anisokaryocytosis and traversing capillaries against a background of hemorrhage. A spindle cell lesion was diagnosed provisionally based on the microscopic findings.

Following the acquisition of informed consent, we planned the surgical intervention for the mass excision. The procedure was conducted under local anesthesia, involving a curvilinear incision around the pedicle of the swelling. The tumor was meticulously dissected from the surrounding structures and excised completely. The wound closure was performed in layers, incorporating the placement of a suction drain. The patient did not report any neural deficits or complications post excision. In the postoperative phase, the patient received intravenous fluids, antibiotics, analgesia, and other symptomatic medications. Regular dressings were done, and the patient was discharged on the fifth day of the postoperative period.

**Figure 2:**
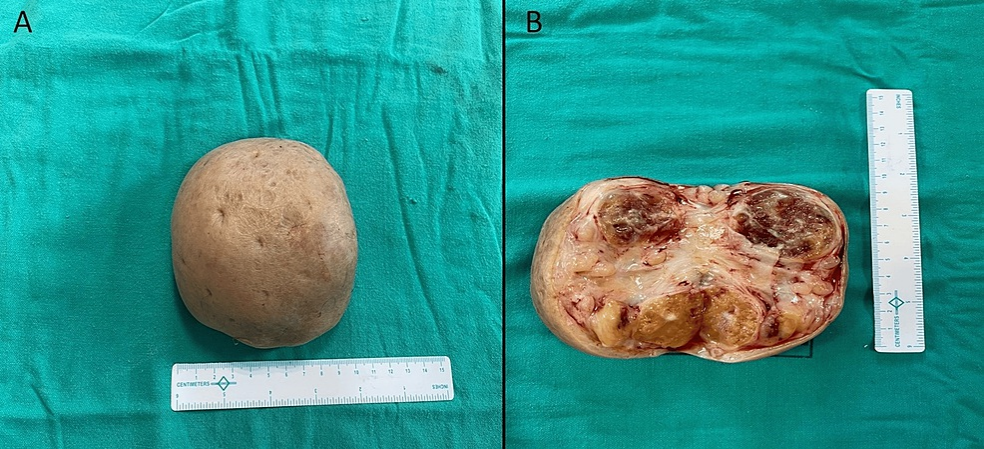
(A) Gross specimen and (B) cut section of the excised mass

The excised mass underwent histopathological examination. The gross analysis revealed a mass measuring 10 × 7 × 7 cm, with the outer surface displaying intact, normal-appearing skin, as depicted in Figure 2.

**Figure 3:**
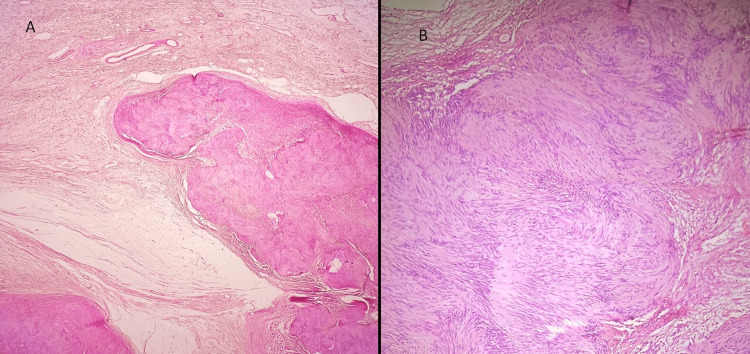
(A) Plexiform nodule of tumor in the dermis and (B) Antoni A areas showing tumor spindle cells exhibiting nuclear palisading and Verocay body formation A: H&E at 20×; B: H&E at 200× H&E: hematoxylin and eosin

No abnormalities were identified on the skin. The cut surface of the mass exhibited multiple discrete, well-defined nodules ranging in size from 0.2 cm to 2.5 cm. These nodules were separated by gelatinous myxoid connective tissue (fibrous septae). The cut surface of the nodules appeared yellow to grey-white, with the presence of microcysts ranging from 0.2 to 0.5 cm in diameter. Some of the larger nodules showed foci of hemorrhage. The microscopic examination of representative sections, as depicted in Figure 3, revealed the skin with deep dermis and subcutaneous adipose tissue exhibiting encapsulated plexiform nodules of a tumor. These nodules displayed varying cellularity with Antoni A and Antoni B areas, comprising spindle cells characterized by elongated nuclei and fibrillary cytoplasm with indistinct cell borders. Nuclear palisading forming Verocay bodies was observed. Some cells exhibited degenerative atypia, manifested by enlarged nuclei and smudged chromatin. The intervening stroma demonstrated edema with cystic change, foam cell change, areas of stromal hyalinization, and thick-walled blood vessels, with some showing perivascular hyalinization. Mitotic activity was not identifiable, and very focal necrosis was observed. Importantly, no evidence of malignancy was found, leading to the diagnosis of plexiform schwannoma.

Suture removal took place two weeks after the surgical procedure. At the one-year follow-up, the patient demonstrated a complete resolution of symptoms, with no observed neurological deficits and no evidence of recurrence. The patient successfully resumed normal daily activities.

## Discussion

PS is a rare neurogenic tumor first described by Harkin in 1978, and its etiology is not yet fully understood [3]. Most PSs are small, with a maximum diameter of approximately 2 cm, and originate from superficial nerves [4]. Schwannomas are intricately associated with several genetic disorders, notably schwannomatosis, neurofibromatosis type 2 (NF2), and Carney complex type 1 (CC1) [5,6].

Due to its rarity, diagnosing schwannomas can be challenging; when presented with soft-tissue swellings in the extremities, it is crucial to consider schwannoma in the differential diagnosis [7]. Magnetic resonance imaging (MRI) stands out as a powerful tool for diagnosing plexiform schwannoma, offering definitive insights. Schwannomas manifest in MRI as fusiform masses with tapered ends, displaying high intensity on T2-weighted images and low to moderate intensity on T1-weighted images [8]. Careful intracranial and spinal MRI is necessary to exclude potential neurofibromatosis type 2 (NF2) in young patients, as up to 5% of PS cases may be associated with NF2 [5-8]. We were not able to get an MRI done as, in resource-limited settings, where MRI is often not feasible due to availability and economic constraints, excisional biopsy becomes a valuable alternative. This approach aided us in achieving an accurate diagnosis, resolving symptoms, and distinguishing swellings from other soft-tissue tumors. Ultrasonography, the primary investigation for superficial swellings, offers a noninvasive and cost-effective solution. Schwannomas typically present as homogenous, hypoechoic masses, revealing proximity to nearby nerves. Features such as posterior acoustic enhancement and internal flow patterns may enhance diagnostic insights [7]. However, the ultrasound finding in our case was suggestive of a neoplastic lesion, so we advised an FNA, which found the lesion to be a spindle cell lesion. So, we planned an excisional surgery for the swelling. Histological examination, the diagnostic gold standard for tumors, revealed the distinctive composition of plexiform schwannomas. Microscopy highlighted Antoni A regions rich in spindle cells and Antoni B regions with loose mucoid stroma [8]. Buoyant S-100 protein immunostaining aids in diagnosing plexiform schwannomas, neurofibroma, and malignant peripheral nerve sheath tumors [9]. The biopsy report of the mass confirmed the definitive diagnosis of PS.

Neglecting soft-tissue masses can lead to excessive tumor growth, disfigurement, metastasis, or death. Patients who fail medical attention may seek care only after experiencing sudden changes in the lesion, such as excessive growth, abnormal bleeding, or pain. Although rare, long-standing cases of schwannoma may transform into malignant tumors [8].

In our case, the patient neglected the PS for 30 years out of fear of lower limb amputation. The patient sought medical attention and underwent a successful surgical excision. Physicians must provide proper education about treatment options and the risks of deferred care to help patients make informed decisions. The adequate recognition of soft-tissue masses is critical to prevent devastating consequences in similar scenarios.

## Conclusions

In conclusion, we report a case of plexiform schwannoma in the medial thigh, representing the largest reported instance to date. Even though it is uncommon in the lower limb, it should be in the surgeon's mind when dealing with a painless swelling. Successful surgical intervention led to complete resolution, highlighting the efficacy of timely treatment in such cases. Imaging, including X-ray and ultrasonography, may not be of much use in making a definitive diagnosis of soft-tissue tumors.
